# Case Report of Taeniasis with Gastric Manifestations on CT and Worm Expulsion Post CT Examination

**DOI:** 10.2174/0115734056430817260324102152

**Published:** 2026-04-08

**Authors:** Yifan Liu, Zha Xi Ci Ding, Si Nan Yong Zong, TianMing Bai, Siqi Li, Tao Jiang

**Affiliations:** 1 Department of Radiology, Chongqing University Fuling Hospital, Chongqing, Fuling 408000, China; 2 Department of Radiology, Mangkang County People's Hospital of Xizang, Xizang, Mangkang 854500, China

**Keywords:** Taeniasis, CT, Raw meat, Albendazole, Upper abdominal pain, Worm expulsion

## Abstract

**Introduction/Background:**

In March 2025, a 31-year-old female patient was admitted to Mangkang County People's Hospital in Tibet, complaining of upper abdominal pain for two days, which worsened after meals. She reported a habit of consuming raw beef. This case is unique as it highlights the diagnostic value of CT imaging in identifying taeniasis, particularly when worms are expelled post-CT examination. Previous imaging studies often lacked specific signs, with many cases only confirmed post-surgery. This case provides a valuable reference for the radiological characteristics of taeniasis.

**Case Presentation:**

The patient presented with upper abdominal pain that worsened after meals. Physical examination revealed a soft abdomen with significant tenderness in the upper abdomen, no rebound tenderness, no muscle tension, no palpable liver or spleen below the costal margin, negative McBurney's sign, negative Murphy's sign, no renal percussion tenderness, negative shifting dullness, normal bowel sounds, and no abnormalities in the anus or rectum. Blood tests showed a white blood cell count of 15.15 x 10^9/L, neutrophil percentage of 92.4%, absolute neutrophil count of 14.0 x 10^9/L, high-sensitivity C-reactive protein of 1.3 mg/L, and amylase of 997.15 U/L. Abdominal CT revealed an irregular, poorly defined mass-like heterogeneous density shadow in the stomach, measuring approximately 83 mm x 38 mm x 51 mm (anteroposterior x transverse x craniocaudal), with an enlarged pancreas and surrounding exudation. After the CT scan, the patient vomited inside the CT room, expelling two white worms. The worms were milky white, ribbon-like, about 200 cm long, with a spoon-shaped scolex, body covered in transverse folds, and a slightly thinner tail. A follow-up CT scan after vomiting showed that the gastric mass had disappeared. Based on the worm morphology, epidemiological history, and laboratory findings, the diagnosis was taeniasis and pancreatitis. The patient was treated with fasting, intravenous administration of H2 receptor antagonists and somatostatin to relieve pancreatitis, and albendazole (2 tablets/day for 3 days) for deworming. Follow-up two months later showed no abnormalities.

**Conclusion:**

This case underscores the importance of considering taeniasis in patients presenting with gastric symptoms and abnormal CT findings. The main takeaway lesson is that prompt recognition and appropriate treatment can lead to successful management of taeniasis, even in unusual presentations. Immediate diagnosis after CT examination and expulsion of worms provides valuable insights into the radiological features of taeniasis.

## 1 INTRODUCTION

Taeniasis is a serious parasitic disease that affects both humans and animals. In China, there are three main species of Taenia that cause human infections: Taenia solium (pork tapeworm), *Taenia saginata* (beef tapeworm), and Taenia asiatica (Asian tapeworm) [1]. Humans become infected with taeniasis by consuming undercooked or raw pork, beef, or liver containing cysticerci, or by accidentally ingesting food contaminated with cysticerci [2]. Adult tapeworms reside in the human small intestine, causing taeniasis, while their larvae (cysticerci) can infest various human tissues, leading to cysticercosis [3]. Currently, taeniasis and cysticercosis remain among the most severe zoonotic foodborne parasitic diseases globally, and are listed by the World Health Organization as one of the top ten most harmful foodborne parasitic diseases [4]. Taeniasis is typically diagnosed through stool examination [5]. In this case, we report a patient who was diagnosed after expelling the tapeworm *via* the mouth following a CT scan.

## 2 CASE PRESENTATION

### 2.1 Case Information

The patient is a 31-year-old Tibetan female farmer who was admitted to the hospital due to upper abdominal pain for two days, which worsened after meals. She reported a habit of consuming raw beef. Physical examination revealed a soft abdomen with significant tenderness in the upper abdomen, no rebound tenderness, no muscle tension, no palpable liver or spleen below the costal margin, negative McBurney's sign, negative Murphy's sign, no renal percussion tenderness, negative shifting dullness, normal bowel sounds, and no abnormalities in the anus or rectum. Blood tests showed a white blood cell count of 15.15 x 10^9/L, neutrophil percentage of 92.4%, absolute neutrophil count of 14.0 x 10^9/L, high-sensitivity C-reactive protein of 1.3 mg/L, and amylase of 997.15 U/L.

### 2.2 Epidemiological Investigation

The patient is a Tibetan individual who resides in a pastoral area. She had a habit of consuming raw beef (3-4 times per week). The patient has no history of consuming raw pork, frog meat, snake meat, or tadpoles, nor do they use raw frog meat to treat wounds.

### 2.3 Diagnostic Methods

**Fig. (1a,b):**
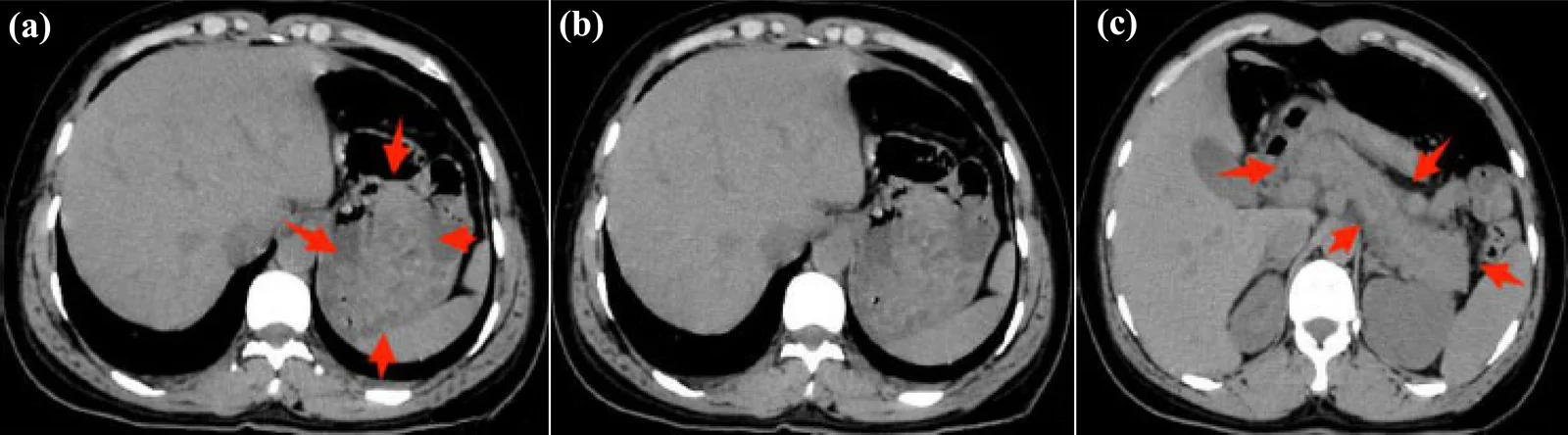
Abdominal CT images showing: A heterogeneous, irregular mass-like density shadow within the stomach, approximately 83 mm x 38 mm x 51 mm (anteroposterior x transverse x craniocaudal), with internal structures resembling curled, ribbon-like objects. The shape is irregular, and the boundaries are poorly defined. **Fig. (1c)** CT image showing an enlarged pancreas with surrounding exudation.

**Fig. (2a-c):**
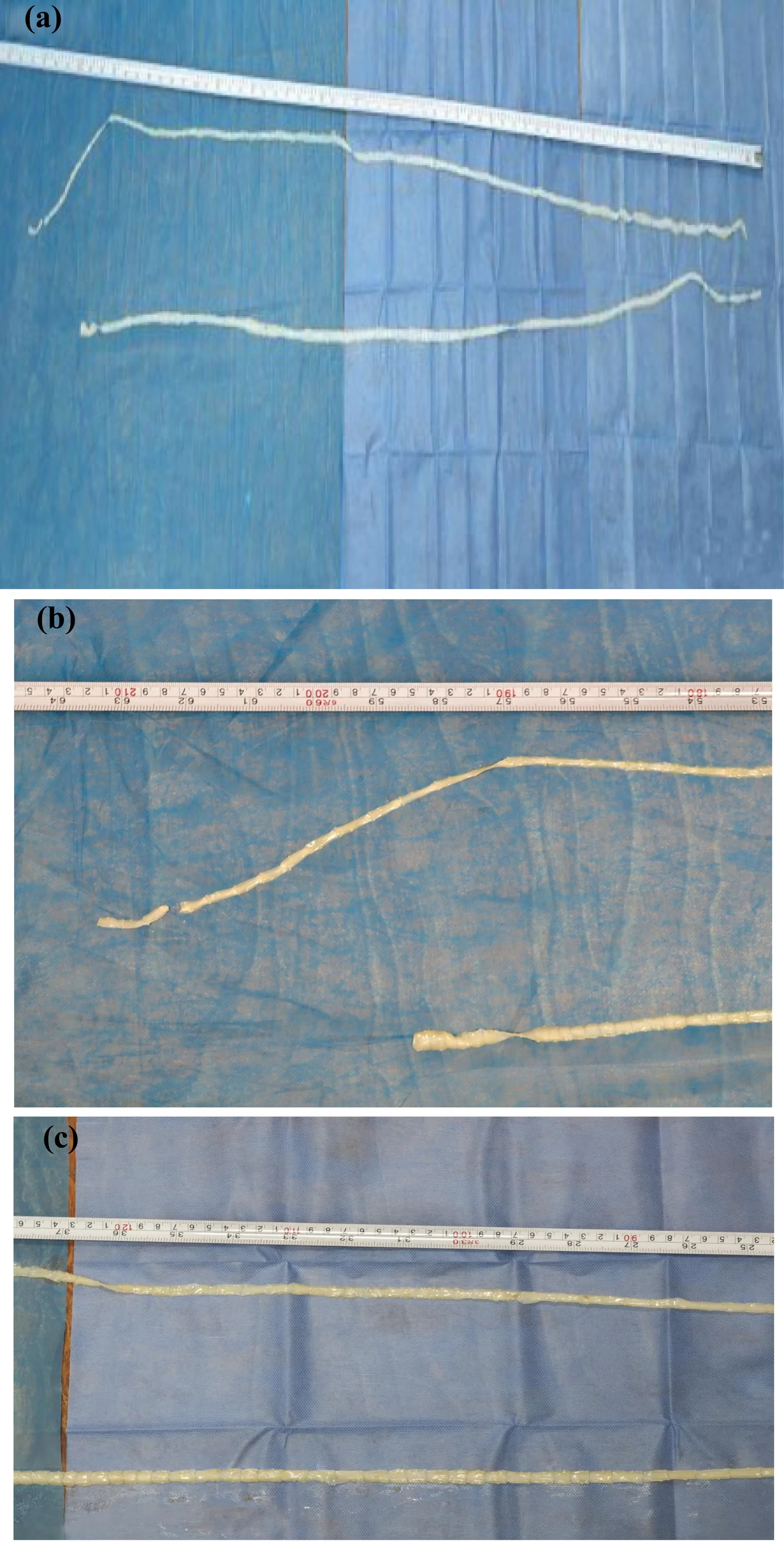
After the CT examination, the patient vomited two white worms.

**Fig. (3):**
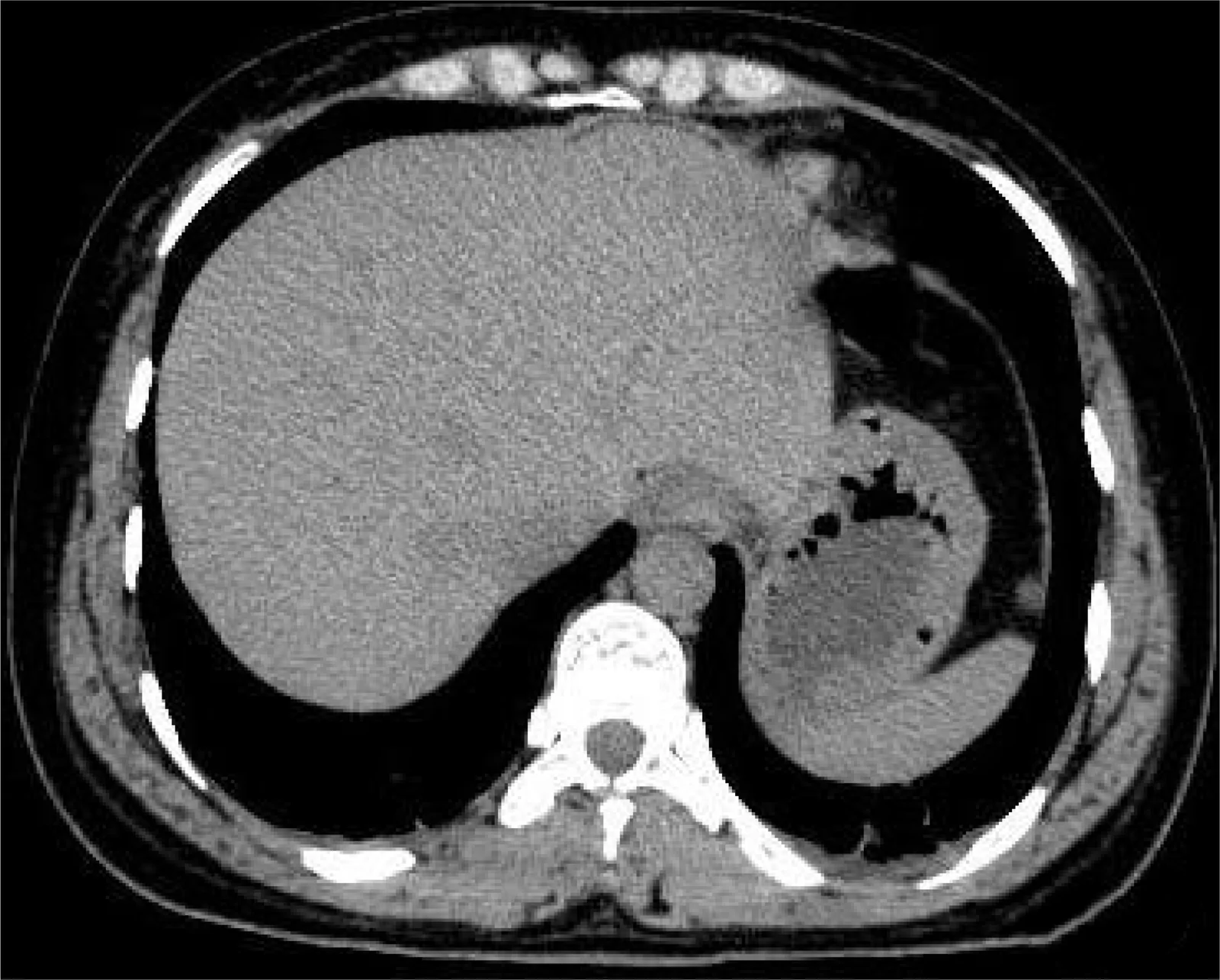
Follow-up abdominal CT shows that the previously observed heterogeneous, irregular mass-like density shadow in the stomach has now disappeared.

Imaging Examination: Abdominal CT of the patient revealed an irregular, poorly defined mass-like heterogeneous density shadow in the stomach, measuring approximately 83 mm x 38 mm x 51 mm (anteroposterior x transverse x craniocaudal), with internal structures resembling curled, ribbon-like objects. The pancreas appeared enlarged with surrounding exudation (Fig. **1a**-**c**). After the CT scan, the patient vomited inside the CT room, expelling two white worms. The worms were milky white, ribbon-like, about 200 cm long, with a spoon-shaped scolex, body covered in transverse folds, and a slightly thinner tail (Fig. **2a**-**c**). A follow-up CT scan after vomiting showed that the gastric mass had disappeared (Fig. **3**). The CT diagnosis considered both pancreatitis and taeniasis.

#### 2.3.1 CT Diagnosis

The CT findings suggest both pancreatitis and taeniasis.

### 2.4 Diagnosis Basis

Based on the patient's epidemiological history, laboratory tests, and CT scan results, a comprehensive analysis was conducted. The patient had a habit of consuming raw beef, showed elevated eosinophil counts on blood tests, and exhibited a heterogeneous mass-like density shadow within the stomach on CT scans. Additionally, the patient vomited tapeworms after the CT examination, with pancreatic swelling and elevated amylase levels. Therefore, the diagnosis was taeniasis accompanied by pancreatitis.

### 2.5 Treatment and Outcome

The patient was treated with fasting, intravenous administration of H2 receptor antagonists, and somatostatin to relieve pancreatitis. Albendazole (2 tablets per day for 3 days) was administered for deworming treatment. Follow-up two months later showed no abnormalities. Morphological examination of the expelled worms revealed two white worms, milky white, ribbon-like, approximately 200 cm long, with a spoon-shaped scolex. The bodies featured transverse folds, and the tails were slightly thinner (Fig. **2a**-**c**). Based on morphological results and the patient's history of consuming raw beef, the preliminary diagnosis was bovine taeniasis accompanied by pancreatitis.

## 3 DISCUSSION

According to the first national survey on human parasitic distribution, taeniasis was once prevalent in 27 provinces in China [6]. After long-term prevention and control measures, the overall infection rate of taeniasis and cysticercosis has been effectively reduced to low levels. However, in regions such as Yunnan, Tibet, and Sichuan, the infection rates remain relatively high, with some local areas reporting infection rates as high as 9.25% [1, 7, 8]. In Tibetan farming areas, traditional pig and cattle rearing methods involve free-range practices, and toilets are often either non-existent or poorly sanitized. Local villagers also have the habit of consuming raw or undercooked pork, beef, and liver, which are significant risk factors for the spread of taeniasis and cysticercosis [9].

In this case, the patient, a herdsman from a Tibetan area, had a habit of consuming raw beef. Following the CT examination, the patient vomited two tapeworms inside the CT room. By comparing the pre- and post-vomiting CT images, the diagnosis of taeniasis was confirmed. The CT findings showed a mass-like density shadow within the stomach, measuring approximately 83 mm x 38 mm x 51 mm (anteroposterior x transverse x craniocaudal), with a CT value ranging from 21 HU to 66 HU. The shape was irregular, and the boundaries were poorly defined. Previous imaging studies of taeniasis lacked specific signs, and many cases were only confirmed post-surgery. However, this case was diagnosed immediately after CT examination when the worms were expelled, providing valuable reference for the imaging characteristics of taeniasis. Due to technical limitations, serological and molecular biology tests such as anti-Spirometra IgG antibody detection were not performed.

For systemic deworming treatment, albendazole (daily dose of 20 mg/kg, divided into two doses for 10 days) and praziquantel (total dose of 120 mg/kg, taken over five days, three times a day) are commonly used. Some literature reports suggest that using areca nut and pumpkin seeds can also effectively expel tapeworms [10]. In this case, the patient was treated with albendazole, and follow-up showed no abnormalities, indicating good treatment efficacy.

Prevention and control of taeniasis should avoid the consumption of high-risk foods such as raw meats, fish roe, and pickled shrimp and crab. Customized preventive and therapeutic measures should be developed for high-risk populations, extensive public health education campaigns should be conducted to change unhealthy eating habits, and rural sanitation infrastructure should be improved to reduce environmental pollution and lower the risk of disease transmission. Regular fecal and serological examinations should be implemented in high-risk areas to facilitate early detection and treatment of infected individuals.

## CONCLUSION

Taeniasis is relatively common in some regions but is typically diagnosed through stool examinations, with very few cases confirmed by CT imaging. Clinicians can utilize this radiological similarity to include this rare finding in the differential diagnosis and appropriate treatment of taeniasis. In the future, we may be able to adopt methods from certain studies [11, 12] to diagnose gastric lesions by analyzing changes in 18F-FDG PET/CT metabolic parameters or by using IoT-connected point-of-care devices.

## Data Availability

The data and supportive information are available within the article.

## ABBREVIATION

CT: Computed Tomography

## STANDARDS OF REPORTING

CARE guidelines were followed.
